# Journal of Balneology

**Published:** 1890-11

**Authors:** 


**Journal of Balneology.** Issued by the Journal of Balneology Publishing Co. New York, P. O. Box 1670. Vol. IV, No. 3, August, 1890.

This is a comparatively new journal, devoted to information concerning natural mineral springs, health resorts, and sanitariums. It is a bi-monthly journal; price, $1 a year, in advance.

In an address to the medical profession the publishers say: "The aim of the journal will be to present each month, in as concise a manner as possible, all available information regarding the therapeutics and the chemical composition of mineral springs, their location, climate, convenience of access, and hotel accommodations. It will also consider the value of thermal springs, the climatology of seaside and inland resorts, their availability for the convenience of the invalid and for the rest and recreation of those who desire to maintain the health and comfort of themselves and families."

The number before us contains an interesting article upon the Hot Springs of Arkansas, besides other interesting articles. If the volume when completed is supplied with a thorough index to all the articles that have been published, it must prove a valuable work of reference. W. M. J.

